# Systematic identification and analysis of dysregulated miRNA and transcription factor feed‐forward loops in hypertrophic cardiomyopathy

**DOI:** 10.1111/jcmm.13928

**Published:** 2018-10-19

**Authors:** Hongbo Shi, Jiayao Li, Qiong Song, Liang Cheng, Haoran Sun, Wenjing Fan, Jianfei Li, Zhenzhen Wang, Guangde Zhang

**Affiliations:** ^1^ College of Bioinformatics Science and Technology Harbin Medical University Harbin China; ^2^ Department of Cardiology The Fourth Affiliated Hospital of Harbin Medical University Harbin China; ^3^ Emergency Cardiovascular Medicine Inner Mongolia Autonomous Region People's Hospital Hohhot China

**Keywords:** feed‐forward loop, miRNA, transcription factor

## Abstract

Hypertrophic cardiomyopathy (HCM) is the most common genetic cardiovascular disease. Although some genes and miRNAs related with HCM have been studied, the molecular regulatory mechanisms between miRNAs and transcription factors (TFs) in HCM have not been systematically elucidated. In this study, we proposed a novel method for identifying dysregulated miRNA‐TF feed‐forward loops (FFLs) by integrating sample matched miRNA and gene expression profiles and experimentally verified interactions of TF‐target gene and miRNA‐target gene. We identified 316 dysregulated miRNA‐TF FFLs in HCM, which were confirmed to be closely related with HCM from various perspectives. Subpathway enrichment analysis demonstrated that the method was outperformed by the existing method. Furthermore, we systematically analysed the global architecture and feature of gene regulation by miRNAs and TFs in HCM, and the FFL composed of hsa‐miR‐17‐5p, FASN and STAT3 was inferred to play critical roles in HCM. Additionally, we identified two panels of biomarkers defined by three TFs (CEBPB, HIF1A, and STAT3) and four miRNAs (hsa‐miR‐155‐5p, hsa‐miR‐17‐5p, hsa‐miR‐20a‐5p, and hsa‐miR‐181a‐5p) in a discovery cohort of 126 samples, which could differentiate HCM patients from healthy controls with better performance. Our work provides HCM‐related dysregulated miRNA‐TF FFLs for further experimental study, and provides candidate biomarkers for HCM diagnosis and treatment.

## 1 INTRODUCTION

Hypertrophic cardiomyopathy (HCM) is the most common genetic cardiovascular disease and is a leading cause of disability and death in patients of all ages, especially sudden and unexpected cardiac death in young people.^1^ From encoding protein RNAs to non‐coding RNAs, our understanding about HCM has improved dramatically both clinically and pathophysiologically. However, the potential molecular mechanisms underlying the pathology of HCM have not been fully understood.

Amongst many genetic factors, miRNAs and transcription factors (TFs) are two types of key gene regulators, and they both participate in many important cellular processes, including cell differentiation, proliferation, and apoptosis.^2^ MiRNAs mainly regulate gene expression at the post‐transcriptional level, while TFs modulate gene transcription at the transcriptional level. Researchers have demonstrated that miRNAs and TFs may synergistically regulate the same target genes, and they may mutually regulate one another; hence forming feed‐forward loops (FFLs), which have been reported to form recurrent network motifs and play important roles in the mammalian gene regulatory network.^2^, ^3^ Thus, dysregulated miRNA‐TF FFLs will lead to a series of diseases, and deciphering the interplay between miRNAs and TFs by means of FFLs will yield new mechanistic insights into specific biological events.

Currently available biological databases have integrated different types of molecular interactions, which made it possible to identify miRNA‐TF FFLs. For instance, TarBase,^4^ miRTarBase,^5^ and miRecords^6^ collected experimentally verified miRNA‐gene regulatory relationships for different organisms, while TargetScan^7^ predicted biological targets for miRNAs. TRED^8^ and Transfac^9^ offered experimentally confirmed TF‐gene regulatory relationships, while JASPAR^10^ predicted TF targets. TransmiR database^11^ recruited experimentally verified TF‐miRNA regulatory pairs. Additionally, large amount of genome‐wide data such as microarray data and next‐generation sequencing data also provide us more valuable information to investigate dysregulated FFLs implicated with specific cellular processes and diseases.

Much efforts have been devoted to detect miRNA‐TF FFLs, which were used to dissect potential regulatory mechanisms underlying human diseases.^12^, ^13^ On the one hand, started from disease‐related molecules and different regulatory relationships amongst miRNAs, genes and TFs, Ye et al^14^ revealed that miR‐19 inhibited CYLD through the identified disrupted FFLs in T‐cell acute lymphoblastic leukaemia. Qin et al^15^ constructed gene regulatory networks involved in miRNA‐TF FFLs for three subtypes of breast cancer, and investigated their distinct and common characteristics. In addition, 4‐node FFLs were proposed and identified by Sun et al^16^ in glioblastoma, from which critical miRNAs and subnetworks were detected. Recently, Zhang et al^12^ and Arora et al^13^ have reviewed miRNA and TF FFLs involved in various biological processes and diseases. On the other hand, computational methods have been developed for identifying dysregulated miRNA and TF FFLs based on sample matched mRNA and miRNA expression profiles.^17^, ^18^ For example, Yan et al^17^ proposed a method, dChip‐GemiNI, for identifying significant miRNA‐TF FFLs associated with five cancers. Subsequently, Jiang et al^18^ developed an algorithm to dissect dysregulated miRNA‐TF FFLs across 13 tumor types, and identified 26 pan‐cancer FFLs. Therefore, using FFLs to decipher the pathological and physiological mechanisms underlying diseases will provide new clues for understanding disease initiation and progression.

In this study, we presented a method for systematically identifying dysregulated miRNA‐TF FFLs in HCM, and 316 dysregulated FFLs were obtained. We found that these dysregulated FFLs were significantly enriched in significantly differentially expressed molecules and the known HCM‐related molecules. Functional analysis also demonstrated that the FFLs were closely associated with HCM. We further investigated the global architecture and feature of regulation between miRNAs and TFs in HCM by constructing a dysregulated miRNA‐TF regulatory network, from which a FFL (hsa‐miR‐17‐5p, STAT3 and FASN) might play important roles in HCM and two panels of diagnostic biomarkers defined by three TFs and four miRNAs were identified.

## 2 MATERIALS AND METHODS

### 2.1 Obtaining and preprocessing of miRNA, gene and TF expression profiles

The HCM related sample‐mathed gene expression profiles and miRNA expression profiles (GSE36961 and GSE36946) were downloaded from the Gene Expression Omnibus database. This expression profiles were obtained by measuring human cardiac tissues from 106 HCM patients and 20 control donors. Protein‐coding genes were retained and miRNA names were manually mapped to standard mature miRNA names based on miRBase database (release 21). If multiple probes corresponded to one gene, the expression values were averaged. A list of human TFs were acquired from a previous study^19^ and TF expression profiles were extracted from gene expression data. Finally, 23 601 mRNAs, 805 miRNAs, 1369 TFs and their corresponding expression profiles were obtained.

### 2.2 Regulatory relationships amongst miRNAs, genes, and TFs

Experimentally verified regulatory relationships amongst miRNAs, genes, and TFs were used. Firstly, experimentally confirmed miRNA‐gene interactions were collected from TarBase (version 6.0),^4^ miRTarBase (version7.0),^5^ and miRecords (version 4)^6^ databases. Secondly, using TFs obtained above, the miRNA‐TF regulatory relationships were determined from miRNA‐gene interactions. Thirdly, experimentally confirmed TF‐gene interactions were collected from TRED^8^ and Transfac,^9^ and experimentally verified TF‐miRNA regulatory relations were retrieved from TransmiR (version 1.2)^11^ database.

### 2.3 Candidate miRNA‐TF FFLs

**Figure 1:**
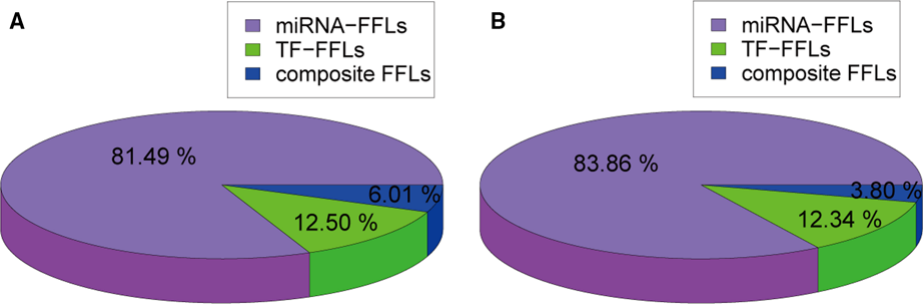
Statistical result of miRNA‐TF FFLs in HCM. (A) The distribution of three types of FFLs in candidate miRNA‐TF FFLs. (B) The distribution of three types of FFLs in dysregulated miRNA‐TF FFLs

In this study, we focused on three‐node miRNA‐TF FFLs, which included a gene, a miRNA, and a TF. According to the main regulator, miRNA‐TF FFLs can be typically classified into three types^12^, ^13^: miRNA‐FFL, TF‐FFL, and composite FFL (Figure S1). In a miRNA‐FFL, miRNA is the main regulator, which controls the expression of a TF and their common target gene, while in a TF‐FFL, TF is the primary regulator. In a composite‐FFL, a miRNA regulates the expression of a TF and a target gene. Simultaneously, the TF dominates the expression of the miRNA and the target gene. By integrating expression profile data and the interactions between miRNAs, TFs, and genes, we identified 6,809 candidate miRNA‐TF FFLs (Figure 1A), which comprised of 5549 miRNA‐FFLs (81.49%), 851 TF‐FFLs (12.50%) and 409 composite FFLs (6.01%). These candidate FFLs included 434 miRNAs, 1253 genes, and 223 TFs.

### 2.4 Identification of dysregulated miRNA‐TF FFLs in HCM

The dysregulated miRNA‐TF FFLs in HCM were identified using sample matched expression profile data. Firstly, each node was scored according to the extent of differential expression^18^, ^20^ using the formulas (1) and (2):

(1) $$S_{\mathrm{node}}=\varphi^{-1}\left(1-2\times \left(1-\varphi \left({\text{Diff}}_{\mathrm{node}}\right)\right)\right)$$

(2) $${\text{Diff}}_{\mathrm{node}}=\left(-\log_{10}p\right)\cdot \left|\log_{2}\text{FC}\right|$$

where φ^−1^ is the inverse normal cumulative distribution function. *p* is the *P*‐value which indicates the significance of differential expression computed by the R “limma” package. FC is the fold change of this gene expression.

Secondly, each edge was scored according to the change of gene co‐expression between HCM case samples and control samples using the following equations^18^, ^21^, ^22^:

(3) $$S_{\mathrm{edge}}=\varphi^{-1}\left(1-2\times (1-\varphi (|X|))\right)$$

(4) $$X=\frac{F\left(r_{\mathrm{case}}\right)\cdot \left(-\log_{10}p_{\mathrm{case}}\right)-F\left(r_{\mathrm{control}}\right)\cdot \left(-\log_{10}p_{\mathrm{control}}\right)}{\sqrt{\frac{1.06}{n_{\mathrm{case}}-3}+\frac{1.06}{n_{\mathrm{control}}-3}}}$$

(5) $$F\left(x\right)=\frac{1}{2}\left(\ln\frac{1+x}{1-x}\right)$$

where *r*_case_ and *r*_control_ are the Spearman correlation coefficient of gene expression in case and control samples, respectively. While *n*_case_ and *n*_control_ represent the samples size, respectively. Function of *F* is Fisher transformation, which have been shown that applying it could improve the power of identifying differentially rewired genes.^23^

Subsequently, the score of a candidate miRNA‐TF FFL was calculated by combining the node score and the edge score as follows:

(6) $$S_{\mathrm{FFL}}=\alpha \frac{\sum_{\mathrm{node}\in \mathrm{FFL}}S_{\mathrm{node}}}{\sqrt{n_{\mathrm{node}}}}+\left(1-\alpha \right)\frac{\sum_{\mathrm{edge}\in \mathrm{FFL}}S_{\mathrm{edge}}}{\sqrt{n_{\mathrm{edge}}}}$$

where *n*_node_ and *n*_edge_ denote the number of nodes and edges in the miRNA‐TF FFL. The parameter α∈(0,1) is used to control the weight of node score and edge score. Here, we considered the node and edge score were weighted equally, and selected α as 0.5.

Finally, a *P*‐value was computed to reflect the significance of a miRNA‐TF FFL. We constructed a random miRNA‐TF FFL by randomly selecting a miRNA, a gene, and a TF, and then calculated the score of this FFL through the above procedure. This process repeated 10 000 times. The empirical *P*‐value was defined as the proportion of randomly obtained FFL scores larger than the real FFL score as below:

(7) $$p\text{‐value}=(\text{Number of}\;S_{\mathrm{random}}>S)/10000$$

In this article, the FFL with *P*‐value <0.01 were selected as dysregulated FFLs.

### 2.5 Collection of genes and miRNAs related to HCM

Genes related to HCM were compiled from a comprehensive human gene‐disease association database, DisGeNET (V5.0),^24^ which integrated many currently widely used gene‐disease databases, such as the Online Mendelian Inheritance in Man database,^25^ the Comparative Toxicogenomics Database,^26^ the Genetic Association Database,^27^ the Mouse Genome Database,^28^ PubMed and Uniprot.^29^ After removing repeating gene‐disease entries, 300 unique genes associated with HCM were obtained.

HCM‐related miRNAs were collected by performing a comprehensive literature review. We searched three manually curated and experimentally confirmed human miRNA‐disease association databases: HMDD (version 2.0),^30^ miR2Disease,^31^ and PhenomiR (February 2011),^32^ and found that none of these databases contained miRNAs related to HCM, although HMDD and miR2Disease included miRNAs associated with cardiac hypertrophy. Therefore, we inquired PubMed using the phrase “hypertrophic cardiomyopathy AND microRNA.” Each article was manually searched for miRNAs with aberrant expression in HCM. The miRNAs were then mapped to standard mature miRNA names from miRBase database (release 21),^33^ and 54 unique mature miRNAs were ultimately selected.

### 2.6 Subpathway enrichment analysis

Existing researches have shown that type‐specific biological functions tend to be located in local areas of the pathway (subpathway) instead of the entire pathway, and thus subpathway may provide more detailed explanations for pathogenesis.^34^, ^35^ KEGG subpathway (local area of the entire biological pathway) enrichment analysis was performed by the R “SubpathwayMiner” package.^34^ Significantly enriched subpathways were identified with a *P* < 0.05. To show the results more clearly, we retained the subpathway with the minimum *P*‐value if multiple significantly enriched subpathways corresponded to an entire pathway.

### 2.7 Identification of potential diagnostic biomarkers in HCM

Potential biomarkers for distinguishing HCM patients from controls were identified using a classification model based on support vector machine (SVM), which was performed using the R “e1071” package. The performance was evaluated by classification accuracy and the area under the receiving operating curve (AUC) using 5‐fold cross‐validation.

The optimal biomarkers in HCM diagnosis were selected using Li et al's method.^36^ We computed classification accuracy for all combinations by applying SVM, and the optimal biomarkers were selected considering a balance between classification accuracy and the number of biomarkers.

## 3 RESULTS

### 3.1 Dysregulated miRNA‐TF FFLs in HCM

We identified 316 dysregulated miRNA‐TF FFLs in HCM using our method (Figure 1B and Table S1). These dysregulated FFLs included 265 miRNA‐FFLs (83.86%), 39 TF‐FFLs (12.34%), and 12 composite FFLs (3.80%). Merging these FFLs 118 miRNAs, 102 genes and 53 TFs were obtained. The number of nodes and links in the FFLs was shown in Table 1.

**Table 1:** Summary of three types of dysregulated miRNA‐TF FFLs in HCM

Motif	Number of FFLs	Number of nodes				Number of links				
		Genes	miRNAs	TFs	Total	miRNA‐gene	miRNA‐TF	TF‐gene	TF‐miRNA	Total
miRNA‐FFL	265	98	101	53	252	232	181	151	–	564
TF‐FFL	39	17	29	11	57	38	–	22	30	90
Composite‐FFL	12	9	5	5	19	10	7	11	7	35
Total	316	102	118	53	273	268	188	171	37	664

### 3.2 Validation of dysregulated miRNA‐TF FFLs for their roles in HCM

We searched for experimentally confirmed miRNA‐TF FFLs in HCM by performing a comprehensive literature review, but no result obtained. Therefore, we validated the dysregulated FFLs from several other perspectives in the absence of gold standard set of FFLs in HCM and compared the method with Jiang et al's method.^18^

**Figure 2:**
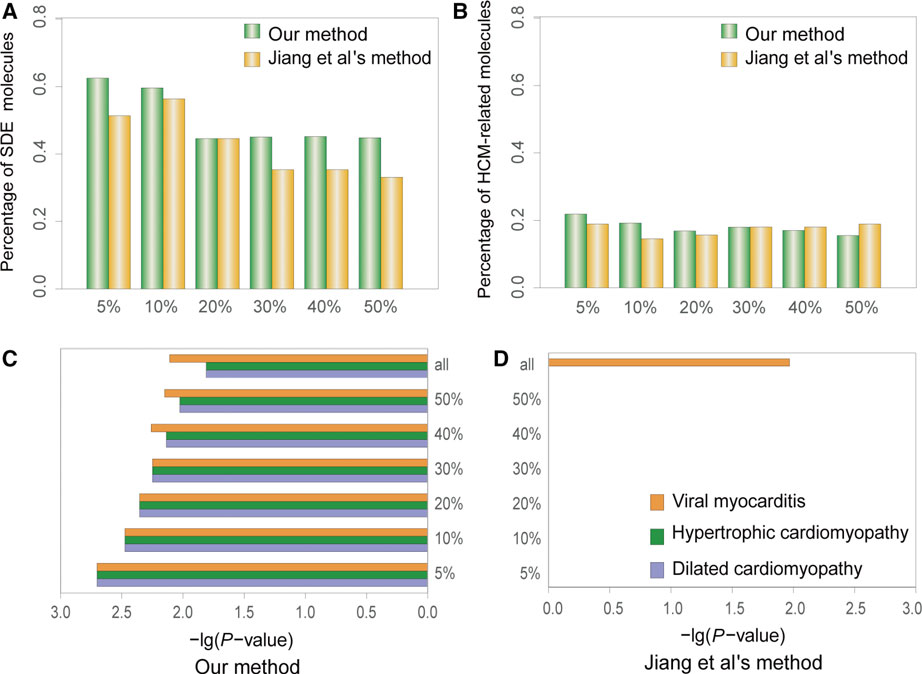
Comparison of our method and Jiang et al.'s method in top 5%, 10%, 20%, 30%, 40%, 50% dysregulated FFLs. (A) The distribution of SDE molecules. (B) The distribution of HCM‐related molecules. (C and D) The distribution of significantly enriched subpathways

We investigated significantly differentially expressed (SDE) miRNAs, genes and TFs in dysregulated FFLs. The R “limma” package with *P* < 0.01 and fold change >1.2 were used to select SDE molecules. As shown in Figure S2, The proportion of SDE molecules was significantly higher than that of candidate FFLs (hypergeometric test, *P* < 0.001). Amongst these SDE molecules, most were down‐regulated. Meanwhile, the proportion of HCM‐related molecules in dysregulated FFLs was significantly higher than candidate FFLs (hypergeometric test, *P* < 0.001). Additionally, SDE molecules and HCM‐related molecules in top 5%, 10%, 20%, 30%, 40%, 50% dysregulated FFLs were examined (Figure 2A and B, Table S2). We observed that the top 5% dysregulated FFLs contained the largest proportion of molecules that were SDE (62.50%) and related to HCM (21.88%). By comparison, the FFLs obtained by Jiang et al's method didn't show this phenomenon.

Biological pathways implicated with dysregulated FFLs were also studied. SubpathwayMiner^34^ was performed to identify significantly enriched KEGG subpathways, and 89 significant subpathways were obtained (*P* < 0.05, Table S3). It is noteworthy that current version of KEGG included five pathways in cardiovascular diseases, and three pathways were significantly enriched: viral myocarditis (*P* = 7.76 × 10^−3^), hypertrophic cardiomyopathy (*P* = 1.55 × 10^−2^), and dilated cardiomyopathy (*P* = 1.55 × 10^−2^). Simultaneously, several other pathways closely related with HCM were identified, including signal transduction‐related PI3K‐Akt and MAPK signalling pathway, inflammation, and immune‐related leucocyte transendothelial migration and T cell receptor signalling pathway, heart function signalling‐related focal adhesion, and axon guidance. We also explored significantly enriched subpathways of top 5%, 10%, 20%, 30%, 40%, 50% dysregulated FFLs. Consequently, they all significantly enriched in the above three cardiovascular diseases pathways, and *P*‐values of the top 5% dysregulated FFLs were the smallest (Figure 2C, Table S3). Compared to Jiang et al's method,^18^ we found that they were not significantly enriched in any cardiovascular diseases pathway, while all the dysregulated FFLs were only enriched in viral myocarditis (Figure 2D, Table S4). All the results demonstrated that the dysregulated FFLs we identified played key roles in HCM, and the more significant the dysregulated FFLs, the more valuable information they will provide for HCM. Additionally, when we limited miRNAs, genes and TFs to be SDE, 19 dysregulated FFLs were obtained. But none of cardiovascular disease pathways was significantly enriched (Table S5), suggesting that the initiation and progression of diseases was caused by not only the expression change of one single gene, but more importantly the inter‐gene interaction.

**Figure 3:**
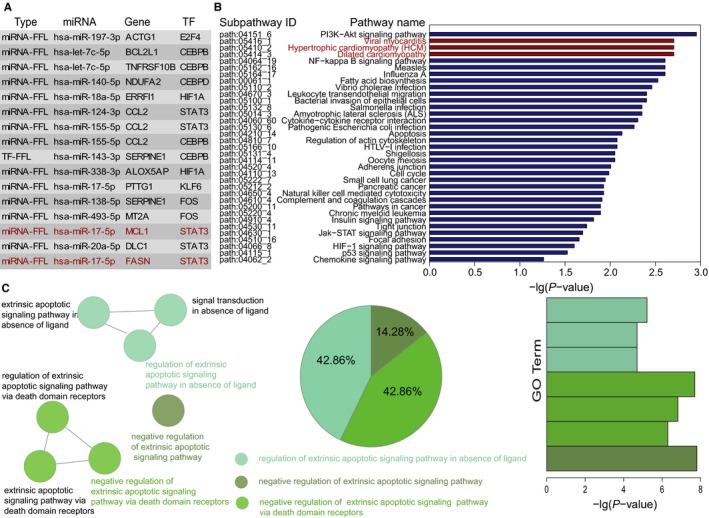
The top 5% dysregulated FFLs and their functional analysis. (A) The top 5% dysregulated FFLs. The red coloured FFLs denote the FFLs mentioned later. (B) Significantly enriched KEGG subpathways. The red coloured pathways denote that they belong to cardiovascular disease pathways in KEGG. (C) Significantly enriched GO terms. The similar GO terms are labelled in the same color

We paid close attention to the top 5% dysregulated FFLs (Figure 3A). These FFLs included 32 molecules, of which 20 were SDE, seven were the known HCM‐related molecules and the remaining 25 molecules were all verified to be implicated with cardiac development or cardiovascular disorders (Table 2 and Table S6). The subpathways enrichment analysis showed that the three cardiovascular disease pathways were all ranked at the top positions (Figure 3B). GO analysis revealed that the genes in top 5% dysregulated FFLs tended to be significantly enriched in three GO terms (Biological Process, *P* < 0.05) functional clusters using Cytoscape plug‐in ClueGO, and these clusters mainly included apoptotic. The known researches showed that apoptosis was involved in the development of myocardial fibrosis in familiar HCM^37^ and apoptosis constituted a major biological phenomenon in the development of HCM.^38^ Interestingly, the FFL formed by hsa‐miR‐17‐5p, MCL1, and STAT3 has been confirmed in a recent report.^39^ Kumar et al showed that miR‐17‐5p regulated autophagy in murine mycobacterium tuberculosis‐infected macrophages by targeting MCL1 and STAT3, and STAT3 silencing suppressed MCL1 levels.^39^ Simultaneously, Xiang et al reported that STAT3 up‐regulated expression of anti‐apoptotic MCL1 in neonatal rat cardiomyocytes.^40^

**Table 2:** Literature validation of nodes in top 5% dysregulated FFLs associated with cardiac development and cardiovascular disorders

Type	Molecule	Known research	Year	PMID
miRNA	hsa‐let‐7c‐5p	(1) Cardiac development	(1) 2017	(1) 29057256
		(2) Cardiac development	(2) 2014	(2) 24365598
		(3) Heart failure	(3) 2016	(3) 27072074
miRNA	hsa‐miR‐124‐3p	(1) Cardiomyocyte hypertrophy	(1) 2017	(1) 28478799
		(2) Vascular smooth muscle cells proliferation and migration	(2) 2017	(2) 29042195
		(3) Atherosclerosis	(3) 2017	(3) 28457624
miRNA	hsa‐miR‐138‐5p	(1) Congenital Heart Disease	(1) 2018	(1) 29298094
		(2) Alcoholic cardiomyopathy	(2) 2015	(2) 25791397
		(3) Cardiac development	(3) 2008	(3) 19004786
miRNA	hsa‐miR‐140‐5p	(1) Cardiac development	(1) 2015	(1) 26465880
		(2) Heart failure	(2) 2016	(2) 27072074
		(3) Cardiotoxicity	(3) 2017	(3) 29304479
miRNA	hsa‐miR‐143‐3p	(1) Dilated cardiomyopathy	(1) 2018	(1) 29335596
		(2) Insulin action in cardiomyocytes	(2) 2013	(2) 23812417
		(3) Coronary heart disease	(3) 2017	(3) 29321799
miRNA	hsa‐miR‐155‐5p	(1) Cardiac hypertrophy	(1) 2015	(1) 26086795
		(2) Dilated cardiomyopathy	(2) 2015	(2) 25840506
miRNA	hsa‐miR‐197‐3p	(1) Cardiometabolic	(1) 2017	(1) 28178938
		(2) Cardiovascular death	(2) 2015	(2) 26720041
miRNA	hsa‐miR‐338‐3p	(1)Diabetic cardiomyopathy	(1) 2014	(1) 23797610
		(2) Autophagy in cardiomyocytes	(2) 2017	(2) 29247537
miRNA	hsa‐miR‐493‐5p	(1) Coronary microembolisation	(1) 2017	(1) 28968594
Gene	ACTG1	(1) Myocardial injury	(1) 2018	(1) 29068691
Gene	ALOX5AP	(1) Familial hypercholesterolemia	(1) 2009	(1) 19361804
		(2) Coronary heart disease	(2) 2010	(2) 21199733
Gene	BCL2L1	(1) Cardiac dysfunction	(1) 2008	(1) 18313710
Gene	CCL2	(1) Cardiomyopathy	(1) 2014	(1) 24980781
		(2) Ischaemic cardiomyopathy	(2) 2007	(2) 17692033
		(3) Cardiac fibrosis	(3) 2009	(3) 19482709
Gene	DLC1	(1) Congenital heart disease	(1) 2014	(1) 24587289
Gene	ERRFI1	(1) Metabolic syndrome	(1) 2016	(1) 27778020
Gene	MCL1	(1) Survival of cardimyocytes during oxidative stress	(1) 2016	(1) 27220418
		(2) Myocardial homoeostasis and autophagy	(1) 2013	(1) 24165322
Gene	MT2A	(1) Cardiomyopathy	(1) 2016	(1) 27477335
		(2) Coronary heart disease	(1) 2014	(2) 25555862
Gene	NDUFA2	(1) Cardiomyocytes oxidative stress	(2) 2013	(1) 23891692
Gene	SERPINE1	(1) Hypertrophic cardiomyopathy	(1) 2013	(1) 23756156
		(2) Heart failure	(2) 2016	(2) 27284354
Gene	TNFRSF10B	(1) Plasma fatty acid distribution	(1) 2010	(1) 20410100
TF	CEBPB	(1) Cardiovascular disease	(1) 2010	(1) 20460359
		(2) Cardiac fibroblast senescence	(2) 2015	(2) 25472717
TF	CEBPD	(1) Ischaemic cardiomyopathy	(1) 2015	(1) 25884818
TF	E2F4	(1) Cardiomyocyte proliferation	(1) 2010	(1) 19955219
		(2) Cardiomyoycte cell proliferation	(2) 2006	(2) 17102628
TF	KLF6	(1) Cardiac fibrosis	(1) 2015	(1) 25987545
		(2) Cardiac fibrosis	(2) 2013	(2) 23724005
TF	STAT3	(1) Cardiac Hypertrophy and Fatty Heart	(1) 2015	(1) 26161779
		(2) Familial hypertrophic cardiomyopathy	(2) 2008	(2) 18362229
		(3) Cardiomyocyte apoptosis	(3) 2014	(3) 25200830

### 3.3 The global architecture and feature of regulation between miRNAs and TFs in HCM

**Figure 4:**
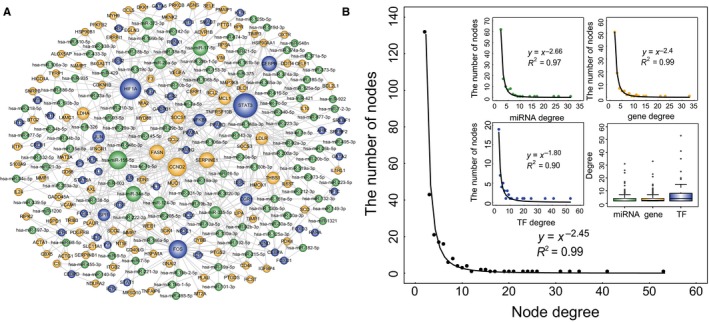
DmiR_TF_Net and its structural features. (A) Global view of the DmiR_TF_Net. The DmiR_TF_Net consists of 664 edges between 118 miRNAs (green circles), 102 genes (yellow circles), and 53 TFs (blue circles). The node size is proportional to the node degree in the network. (B) Degree distribution of all nodes in the DmiR_TF_Net, and degree distribution of miRNAs, genes, and TFs

To investigate the global architecture and feature of gene regulation by miRNAs and TFs in HCM, we constructed dysregulated miRNA‐TF regulatory network (DmiR_TF_Net) via merging three types of dysregulated FFLs identified above. As shown in Figure 4A, the network included 273 nodes (118 miRNAs, 102 genes and 53 TFs) and 664 edges. Amongst these edges, 268 belonged to miRNA‐gene pairs, 188 belonged to miRNA‐TF pairs, 171 belonged to TF‐gene pairs, and 37 belonged to TF‐miRNA pairs.

To examine the global view of the DmiR_TF_Net, we calculated degree and their distribution. Like many other biological networks, degree distribution of this network displayed a power law with a slope of −2.45 and an *R* ^2^ of 0.99, indicating that the network was scale‐free (Figure 4B). Therefore, we observed that most nodes degree was low and only a few nodes highly connected with other nodes. Additionally, the individual degree distribution of miRNAs, genes and TFs also demonstrated the same features. The average node degree of miRNAs, genes, and TFs was 4.18 (range 2‐31), 4.30 (range 2‐33), and 7.47 (range 2‐53), respectively (Figure 4B).

**Figure 5:**
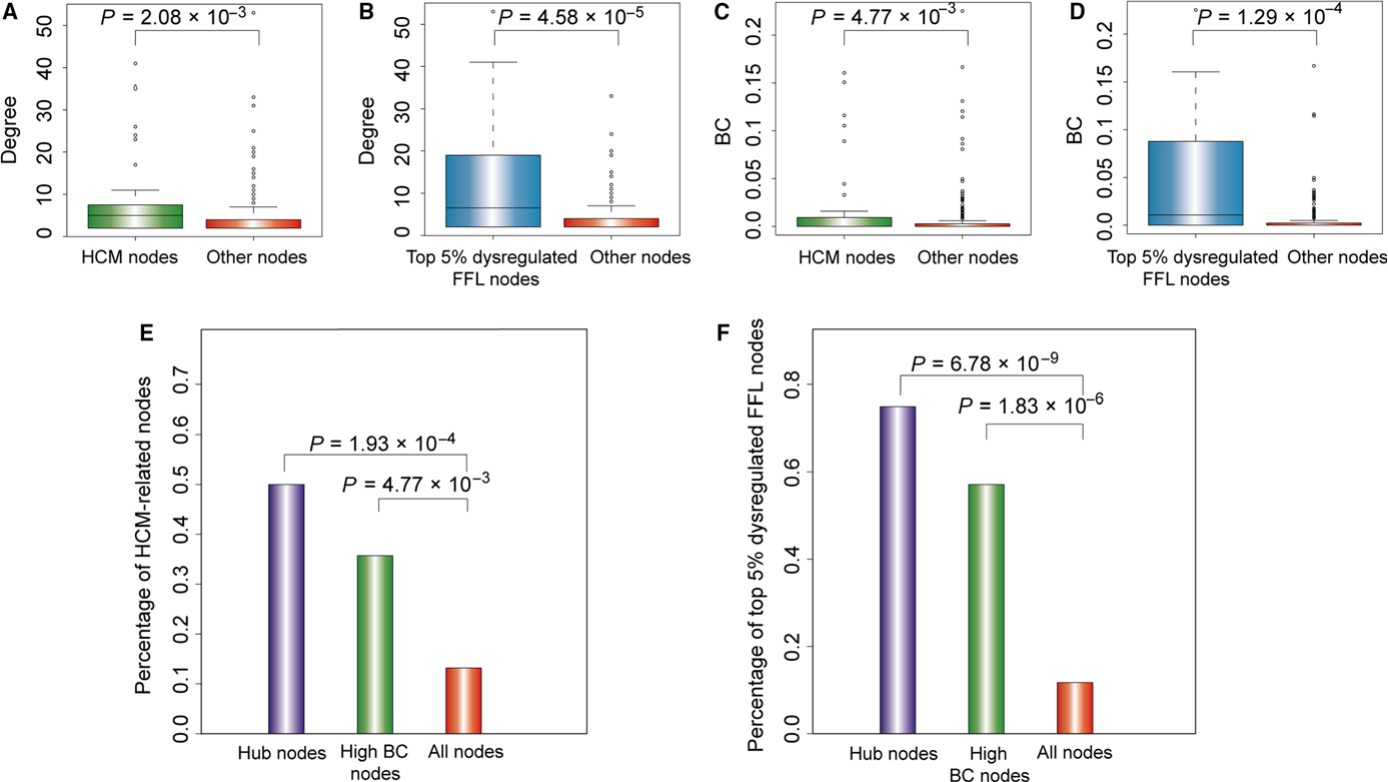
HCM‐related nodes and nodes in top 5% dysregulated FFLs tend to be hubs and high BC nodes. (A and B) The degree of HCM‐related nodes and nodes in top 5% dysregulated FFLs were significantly higher than that of other nodes. (C and D) The BC of HCM‐related nodes and nodes in top 5% dysregulated FFLs was significantly higher than that of other nodes. (E) The percentage of HCM‐related nodes in hub nodes, high BC nodes, and all nodes. (F) The percentage of top 5% dysregulated FFL nodes in hub nodes, high BC nodes, and all nodes

We further investigated degree and betweenness centrality (BC) of the known HCM‐related nodes and nodes in top 5% dysregulated FFLs. As shown in Figure 5A‐D, HCM‐related nodes and top 5% dysregulated FFLs nodes have significantly higher degree and BC than other nodes (Wilcoxon rank sum test). The nodes with highly connected features (hubs) and high BC are usually considered to play critical roles in maintaining the overall connectivity of the network. We selected hub nodes using the method proposed by Yu et al,^41^ and four hub miRNAs (hsa‐miR‐155‐5p, hsa‐miR‐17‐5p, hsa‐miR‐34a‐5p, and hsa‐miR‐20a‐5p), four hub genes (CCND2, FASN, SERPINE1, LDLR) and four hub TFs (STAT3, HIF1A, FOS, and CEBPB) were identified (Table 3). The top 5% miRNAs and TFs with the highest BC were six miRNAs (hsa‐miR‐182‐5p, hsa‐miR‐155‐3p, hsa‐miR‐124‐3p, hsa‐miR‐34a‐5p, hsa‐miR‐17‐5p, and hsa‐miR‐181a‐5p), five genes (PDK4, CCND2, FASN, SERPINE1, and SCD) and three TFs (STAT3, HIF1A, and FOS) (Table 4). Additionally, we observed that the hub nodes and the nodes with high BC were both significantly enriched in nodes related with HCM and nodes within top 5% dysregulated FFLs (Hypergeometric test, Figure 5E and F). All the observations demonstrated that the nodes with high degree and BC might play critical roles in HCM.

**Table 3:** Hub miRNAs, hub genes, and hub TFs in the DmiR_TF_Net in HCM

miRNAs	Degree	Genes	Degree	TFs	Degree
hsa‐miR‐155‐5p^∆^	31	CCND2	33	STAT3^∆^	53
hsa‐let‐17‐5p^#∆^	26	FASN^#∆^	23	HIF1A^#∆^	41
hsa‐miR‐34a‐5p^#^	24	SERPINE1^∆^	21	FOS^#∆^	35
hsa‐miR‐20a‐5p^#∆^	17	LDLR	15	CEBPB^∆^	25

miRNAs, genes and TFs with “^∆^” denote the miRNAs, genes and TFs are within top 5% dysregulated FFLs, and those with “^#^” indicate the known miRNAs, genes and TFs associated with HCM.

**Table 4:** MiRNAs, genes, and TFs with the highest (top 5%) betweenness centrality (BC) in the DmiR_TF_Net in HCM

miRNAs	BC	Genes	BC	TFs	BC
hsa‐miR‐182‐5p	0.1667	PDK4	0.1667	STAT3^∆^	0.2252
hsa‐miR‐155‐5p^∆^	0.1311	CCND2	0.1144	HIF1A^#∆^	0.1605
hsa‐miR‐124‐3p^∆^	0.1205	FASN^#∆^	0.1053	FOS^#∆^	0.1508
hsa‐miR‐34a‐5p^#^	0.1161	SERPINE1^∆^	0.0916		
hsa‐miR‐17‐5p^#∆^	0.0891	SCD	0.0369		
hsa‐miR‐181a‐5p	0.0496				

miRNAs and TFs with “^∆^” denote the miRNAs, genes, and TFs are within top 5% dysregulated FFLs, and those with “^#^” indicate the known miRNAs, genes, and TFs associated with HCM.

Amongst these hub nodes and high BC nodes, we found that hsa‐miR‐17‐5p, FASN, and STAT3 formed a FFL, and this FFL was within the top 5% dysregulated FFLs (Figure 3). Hsa‐miR‐17‐5p and FASN were both known HCM‐related genes. While increased levels of phosphorylated STAT3 were observed in a double‐mutation mouse model of familial HCM, and corresponded with the occurrence of disease.^42^ Additionally, it has been reported that by targeting STAT3, miR‐17‐5p regulated mouse cardiomyocyte apoptosis in response to ischaemia followed by reperfusion^43^ and induced protective autophagy and anti‐apoptosis in vascular smooth muscle cells.^44^

### 3.4 Potential diagnostic biomarkers in HCM

**Figure 6:**
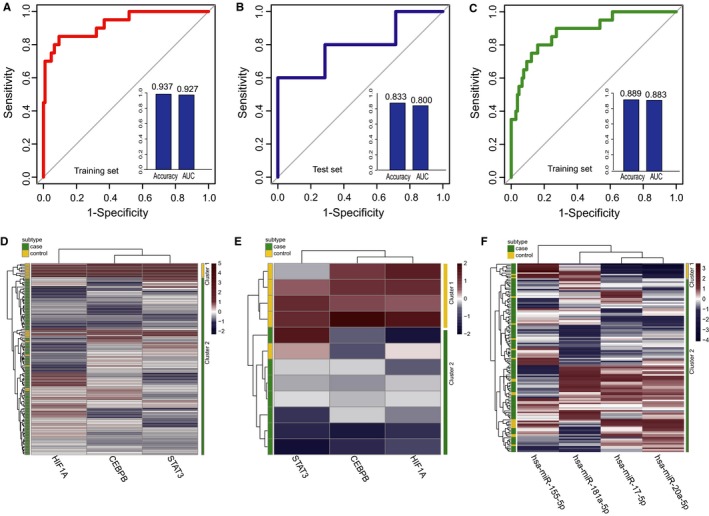
Classification performance of the identified two panel diagnostic biomarkers defined by TFs and miRNAs in HCM based on 5‐fold cross‐validation analysis. (A and B) Performance evaluation of three TFs diagnostic biomarkers in the training set and test set, respectively. (C) Performance evaluation of four miRNAs diagnostic biomarkers in training set. (D and F) The hierarchical clustering heat map of 126 samples based on expression profiles of three TFs and four miRNAs in training set, respectively. (E) Hierarchical clustering heat map of 12 samples based on expression profiles of three TFs in test set

Based on the above observations, we focused on the 12 regulators (five TFs and seven miRNAs) with high degree and BC in the DmiR_TF_Net, which might be associated with HCM occurrence. There were 2^5^ − 1 = 31 and 2^7^ − 1 = 127 combinations of these TFs and miRNAs, respectively. We calculated classification accuracies for all the combinations by applying SVM classification model, and the optimal biomarkers were achieved. Finally, two panel biomarkers defined by three TFs (CEBPB, HIF1A and STAT3) and four miRNAs (hsa‐miR‐155‐5p, hsa‐miR‐17‐5p, hsa‐miR‐20a‐5p, and hsa‐miR‐181a‐5p) with the highest classification accuracy were identified. For the signature of three TFs, an accuracy of 0.937 and an AUC value of 0.927 were obtained in the training set based on 5‐fold cross‐validation (Figure 6A). We further examined this signature in an independent test set (GSE68316) including seven HCM patients and five healthy individuals, and an accuracy of 0.833 and an AUC value of 0.800 were obtained (Figure 6B). Similarly, for the signature of four miRNAs, we achieved an accuracy of 0.889 and an AUC value of 0.883 in the training set (Figure 6C). Hierarchical clustering analysis was performed using expression data of the two panel biomarkers and two major sample clusters with clear differences were found (Figure 6D‐F). For three TFs, the rates of HCM patients in the predicted HCM group were 100% both in training set (106/106) and test set (7/7), whereas the corresponding rates in the predicted healthy group were 45% (9/20) and 80% (4/5), respectively. For four miRNAs, the rates of HCM patients in the predicted HCM group were 92.45% (98/106) in training set, whereas the corresponding rates in the predicted healthy group were 10% (2/20). All these results indicated that the signatures we identified had better performance in distinguishing HCM patients from controls.

## 4 DISCUSSION

TFs and miRNAs are two key regulators that mainly regulate gene expression at the transcriptional and post‐transcriptional level. Accumulating evidence has demonstrated the important roles of their combinational regulation acting as FFLs in various cellular processes and diseases. In this study, we introduced a novel analysis approach for identifying dysregulated miRNA‐TF FFLs between two biological states such as health and disease. We then identified dysregulated miRNA‐TF FFLs associated with HCM, which were confirmed to be closely related with HCM from different perspectives. We investigated the global architecture and feature of dysregulated FFLs in HCM, from which a FFL (hsa‐miR‐17‐5p, STAT3 and FASN) might play important roles in HCM and two panels of diagnostic biomarkers defined by three TFs and four miRNAs were identified.

The main idea of the computational method we proposed is differential co‐expression in normal and disease states. For a miRNA‐TF FFL, we evaluated the strength of its dysregulation by integrating differential expression of the nodes and differential co‐expression of the edges in the FFL. Therefore, differential co‐expression analysis can not only reflect dynamic changes of a single gene, but also capture the connections between genes. This approach was originated from Jiang's method,^18^ and we improved it. Compared with Jiang's method, we observed that the more significant the FFLs we identified, the more HCM‐related biological information they contained. The investigation of biological pathway also revealed that the dysregulated FFLs we identified were significantly enriched in three cardiovascular disease pathways including HCM pathway, while Jiang's method^18^ only enriched in viral myocarditis.

More importantly, the dysregulated FFLs we identified provide important clues for further experimentally validation and studying combinational regulation of miRNAs and TFs in HCM. The dysregulated FFLs were based on experimentally verified regulatory relationships among miRNAs, genes, and TFs. Here, we focused more on the accuracy than the coverage, and thus the computational predicted interactions were not used. However, it is neither complete nor unbiased for experimentally confirmed data, and many regulatory relationships among miRNAs, genes, and TFs were not verified in HCM‐related cells or tissues. Therefore, further experimental confirmation was warranted. With an improvement of the quantity and quality of these data, the dysregulated miRNA‐TF FFLs we identified will be more accurate and comprehensive.

In summary, we systematically identified dysregulated miRNA‐TF FFLs in HCM, and confirmed their important roles in HCM. Our results provide important clues for investigation of gene regulation by miRNAs and TFs in HCM, and shed new light on deciphering the pathogenesis of HCM at the transcriptional and post‐transcriptional levels.

## CONFLICT OF INTEREST

The authors declare no conflict of interest.
