# Supplementary figures and images for: Systematic identification and analysis of dysregulated miRNA and transcription factor feed‐forward loops in hypertrophic cardiomyopathy
Source: J Cell Mol Med. 2018 Oct 19;23(1):306–16. doi: 10.1111/jcmm.13928 (PMC6307764; doi:10.1111/jcmm.13928)

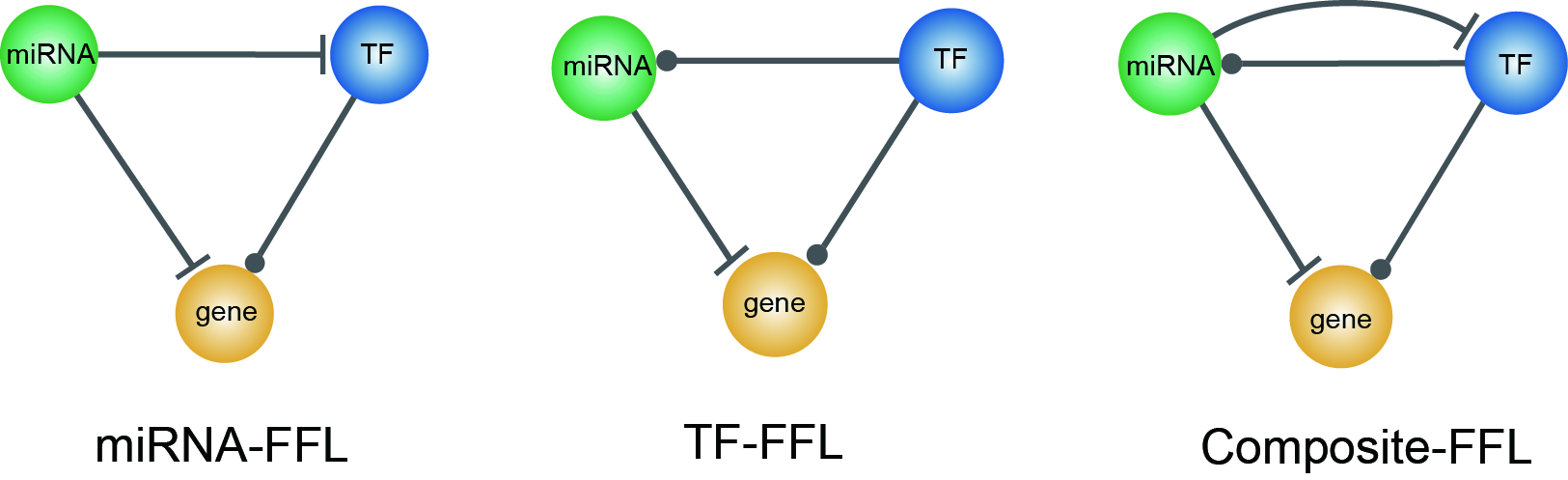

Supplement: Supplementary file 1 [file JCMM-23-306-s001.tif]

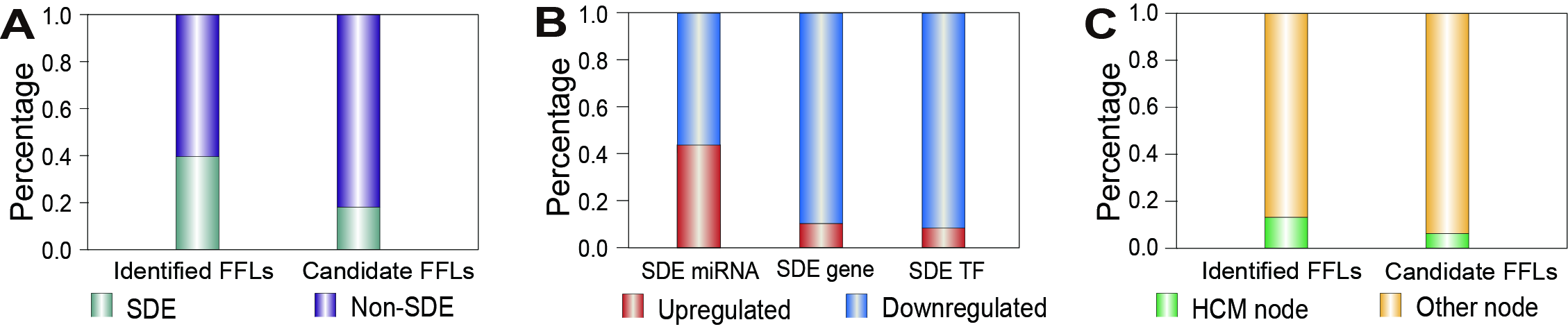

Supplement: Supplementary file 2 [file JCMM-23-306-s002.tif]
