# Supplementary material for: Systematic identification and analysis of dysregulated miRNA and transcription factor feed‐forward loops in hypertrophic cardiomyopathy
Source: J Cell Mol Med. 2018 Oct 19;23(1):306–16. doi: 10.1111/jcmm.13928 (PMC6307764; doi:10.1111/jcmm.13928)
Supplement: Supplementary file 4 [file JCMM-23-306-s004.docx]

## Supplementary Table S2. The percentage of SDE molecules (miRNAs, genes and TFs) and the known HCM-related molecules in top 5%, 10%, 20%, 30%, 40%, 50% dysregulated FFLs obtained using our method and Jiang et al.'s method.

|  | **Top 5%** | **Top 10%** | **Top 20%** | **Top 30%** | **Top 40%** | **Top 50%** |
| --- | --- | --- | --- | --- | --- | --- |
| **SDE_our** | 62.50% | 59.62% | 44.58% | 45.05% | 45.19% | 44.83% |
| **SDE_Jiang's** | 51.35% | 56.36% | 44.58% | 35.34% | 35.34% | 33.11% |
| **HCM_our** | 21.88% | 19.23% | 16.87% | 18.02% | 17.04% | 15.52% |
| **HCM_Jiang's** | 18.92% | 14.55% | 15.66% | 18.05% | 18.05% | 18.92% |
